# Revealing the Origin of Low‐Temperature Activity of Ni–Rh Nanostructures during CO Oxidation Reaction with Operando TEM

**DOI:** 10.1002/advs.202105599

**Published:** 2022-05-05

**Authors:** Tanmay Ghosh, Xiangwen Liu, Wenming Sun, Meiqi Chen, Yuxi Liu, Yadong Li, Utkur Mirsaidov

**Affiliations:** ^1^ Department of Physics National University of Singapore Singapore 117551 Singapore; ^2^ Centre for BioImaging Sciences Department of Biological Sciences National University of Singapore Singapore 117557 Singapore; ^3^ Institute of Analysis and Testing Beijing Academy of Science and Technology (Beijing Center for Physical and Chemical Analysis) Beijing 100094 P. R. China; ^4^ College of Science China Agricultural University Beijing 100193 P. R. China; ^5^ College of Environmental and Energy Engineering Beijing University of Technology Beijing 100124 P. R. China; ^6^ Department of Chemistry Tsinghua University Beijing 100084 P. R. China; ^7^ Centre for Advanced 2D Materials and Graphene Research Centre National University of Singapore Singapore 117546 Singapore; ^8^ Department of Materials Science and Engineering National University of Singapore Singapore 117575 Singapore

**Keywords:** CO oxidation, heterogeneous catalysis, nanoparticles, operando TEM

## Abstract

In bimetallic heterostructured nanoparticles (NPs), the synergistic effect between their different metallic components leads to higher catalytic activity compared to the activity of the individual components. However, how the dynamic changes through which these NPs adopt catalytically active structures during a reaction and how the restructuring affects their activity are largely unknown. Here, using operando transmission electron microscopy, structural changes are studied in bimetallic Ni–Rh NPs, comprising of a Ni core whose surface is decorated with smaller Rh NPs, during a CO oxidation reaction. The direct atomic‐scale imaging reveals that, under O_2_‐rich conditions, Ni core partially transforms into NiO, forming a (Ni+NiO)–Rh hollow nanocatalyst with high catalytic activity. Under O_2_‐poor conditions, Rh NPs alloy with the surface of the core to form a NiRh‐alloy surface, and the NPs display significantly lower activity. The theoretical calculations indicate that NiO component that forms only under O_2_‐rich conditions enhances the activity by preventing the CO poisoning of the nanocatalysts. The results demonstrate that visualizing the structural changes during reactions is indispensable in identifying the origin of catalytic activity. These insights into the dynamic restructuring of NP catalysts under a reactive environment are critical for the rational design of high‐performance nanocatalysts.

## Graphical abstract

Using operando transmission electron microscopy, this study tracks individual bimetallic Ni–Rh nanoparticles (NPs) during a CO oxidation reaction in real‐time and reveals the details of the NPs’ dynamic structure responsible for their low‐temperature catalytic activity. Namely, the interface between Rh and newly formed NiO significantly reduces the CO poisoning of Rh at low temperatures boosting its activity.

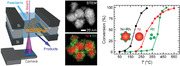

## 1 Introduction

Bimetallic heterostructured nanoparticles (NPs) are an important class of catalysts because by exploiting the synergy between two metals, they can catalyze reactions significantly better than their individual components.^[^ ^1^, ^2^, ^3^, ^4^ ^]^ The rational design of these nanocatalysts requires a detailed understanding of the catalytically active sites, which is determined by their nanoscale structure and chemical state under reaction conditions. Probing these nanocatalysts to identify the relationship between their structure and activity is challenging because their structure is dynamic and easily altered by the interaction with reactants that continuously adsorb onto and products that desorb from the surface, which in turn modifies the catalytic activity during the reaction.^[^ ^5^, ^6^, ^7^ ^]^ For example, under CO gas environment, Pt NPs restructure such that their {100} surface facets are dominated by high‐index steps.^[^ ^7^ ^]^ Another example would be the oxygen‐induced formation of nanosized {112} facets on MgO‐supported Pd NPs.^[^ ^8^ ^]^ The interaction between adsorbates and surfaces is more complicated for bimetallic heterostructured NPs than for monometallic NPs because individual components and interface between these components all interact differently with the adsorbates. Hence, an approach that enables to directly probe the detailed structure of the bimetallic NPs and correlate the structure with the activity under the reaction condition is critical in revealing the origin of their synergetic activity.^[^ ^5^, ^9^, ^10^ ^]^

The adsorption and oxidation of CO molecules over noble metals are the basic surface processes that are commonly used to probe heterogeneous catalysis.^[^ ^11^, ^12^, ^13^, ^14^ ^]^ Thus far, operando studies, such as scanning probe microscopy, infrared absorption spectroscopy, synchrotron X‐ray‐based methods, and X‐ray photoelectron spectroscopy, mostly focus on using bulk single crystal model catalysts to extrapolate the key structure–property relationships.^[^ ^15^, ^16^, ^17^, ^18^ ^]^ However, the structural changes in bulk catalysts are not necessarily similar to those of NPs that are widely used in current technologies.^[^ ^8^, ^10^, ^19^ ^]^ Even when supported NP catalysts are employed, the measurements are limited to their averaged behavior,^[^ ^20^, ^21^ ^]^ making it challenging to discern between chemical or morphological effects that are responsible for their catalytic performance. Hence, tracking the evolution of individual NP catalysts under reactive environments is critical in establishing the origin of their activity. The dynamic structure of NPs during reactions can be explored with atomic‐scale details during reactions using operando transmission electron microscopy (TEM) because this approach enables to both measure catalytic performance and follow the transformation of individual nanocatalysts during the reaction under relevant reaction conditions (i.e., atmospheric gas pressure, relevant pressure ratios, and temperatures).^[^ ^10^, ^22^, ^23^ ^]^

## 2 Results and Discussion

**Figure 1:**
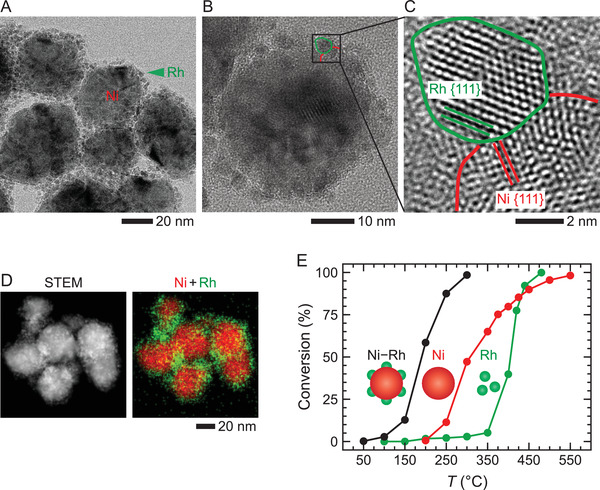
Morphology and CO oxidation activity of Ni–Rh heterostructured NPs. A) Low and B) high magnification TEM images of Ni–Rh heterostructured NPs. C) High‐resolution TEM image from the area selected by the black box in (B). Green and red curves highlight the boundaries of Rh and Ni components, respectively. The lattice spacings of 0.20 and 0.22 nm correspond to Ni {111} and Rh {111}, respectively. D) STEM image and corresponding EDX chemical maps of Ni–Rh heterostructured NPs. E) Temperature‐dependent performance of the Ni–Rh NPs in CO oxidation reaction.

Here, we used operando TEM to study the structural and compositional changes in strawberry‐shaped Ni–Rh heterostructured NPs shown in **Figure** **1**A–D during a CO oxidation reaction. We chose to use noble metal Rh and non‐noble metal Ni to build bimetallic heterostructured NPs because both are considered to have good catalytic performance for CO oxidation.^[^ ^24^, ^25^, ^26^ ^]^ The catalyst consists of a bigger size Ni NP (≈ 40–80 nm) whose surface is decorated with small (≈ 3–5 nm) Rh NPs. This configuration of NP allows us to reduce the amount of precious Rh in the catalyst NPs. Our direct imaging of the Ni–Rh NPs combined with simultaneous monitoring of CO_2_ conversion rate during the CO oxidation reaction show that new surface structures can form under different reactive environments, resulting in different CO oxidation activities.

Our Ni–Rh heterostructured NPs shown in Figure 1 were produced using a new one‐pot synthesis protocol (see the Experimental Section). These NPs comprise of 40–80 nm Ni core uniformly decorated with numerous 3–5 nm Rh NPs (Figure 1A,B). The lattice spacings of 0.20 and 0.22 nm, measured from the high‐resolution TEM images, matching with Ni {111} and Rh {111} planes, respectively, confirm the crystallinity of the central Ni and peripheral Rh NPs (Figure S1, Supporting Information). The well‐defined interface between Ni and Rh lattices, as shown in Figure 1C, suggests that the Rh NPs have directly grown on the surface of Ni NPs. The high‐angle annular dark‐field scanning TEM (STEM) imaging and energy‐dispersive X‐ray spectroscopy (EDX) mapping (Figure 1D) show that almost all Rh NPs are located on the surface of core Ni NPs, further confirming that Rh NPs grow directly on the core.

We evaluated the catalytic performance of the Ni–Rh, pure Ni, and pure Rh NPs for the CO oxidation reaction in a conventional flow reactor (see the Experimental Section for details). Our results show that these Ni–Rh NPs have a much lower activation temperature than that of pure Ni and Rh NPs (Figure 1E), indicating that the synergy is responsible for this significantly improved performance.

**Figure 2:**
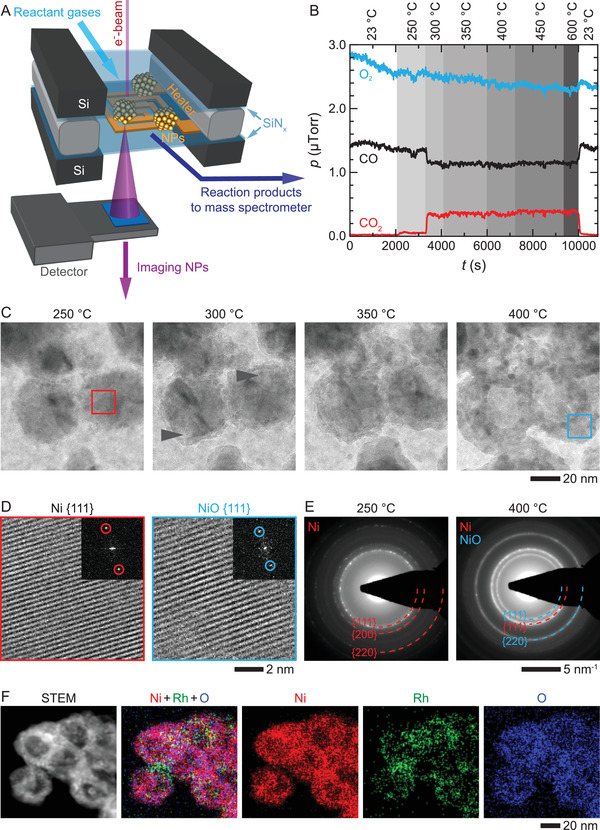
Operando transmission electron microscopy (TEM) of Ni–Rh NPs during CO oxidation. A) Schematic of the experimental setup where Ni–Rh NPs are encapsulated within a microfabricated gas‐cell nanoreactor with an integrated thin‐film heater. The reactant and product gases are analyzed using an inline mass spectrometer. B) Changes in gas compositions during the CO oxidation reaction at different temperatures under 760 Torr of 9% CO, 18% O_2_, and 73% He gas environment, which corresponds to a gas pressure ratio of $p_{\mathrm{CO}}/p_{O_{2}}\approx 0.5$. C) Sequence of TEM images of Ni–Rh NPs at elevated temperatures and under the gas environment described in (B). Black arrows indicate starting of the hollowing. D) High‐resolution TEM images from the areas selected by red and blue boxes in (C). Insets are the corresponding FFT patterns. The lattice spacing of *d*_Ni {111}_ =  0.20 nm changed to *d*_NiO {111}_ =  0.24 nm when the temperature was increased from 250 to 400 °C. E) Electron diffraction patterns of Ni–Rh NPs at 250 and 400 °C, and dashed red and blue quarter rings correspond to Ni and NiO planes, respectively. Note that because of an extremely small amount of Rh, diffraction rings associated with Rh are absent. F) STEM image and corresponding EDX chemical maps of Ni–Rh NPs after the reaction at 400 °C.

To identify the origin of the synergetic enhancement of catalytic performance, we image the changes in the structures and compositions of Ni–Rh heterostructured NPs during the CO oxidation reaction using an in situ gas‐phase TEM platform shown in **Figure** **2**A and described in our earlier reports.^[^ ^5^, ^6^ ^]^ This setup enables to simultaneously image nanocatalysts and analyze the product gasses at different temperatures using an inline mass spectrometer connected to the output of the gas‐cell nanoreactor. We introduced the reaction gas mixture of O_2_ (mixed with 80% He) and CO into the microfabricated nanoreactors and monitored the catalytic activity and structural change in the NPs at various reaction conditions (i.e., temperatures and $p_{\mathrm{CO}}/p_{O_{2}}$ partial pressure ratios).

To test how the reactant gases affect the Ni–Rh NPs, we performed operando studies under two different (O_2_‐rich and O_2_‐poor) environments. We obtained the O_2_‐rich environment by introducing a gas mixture comprising 9% CO, 18% O_2_, and 73% He $\left(p_{\mathrm{CO}}/p_{O_{2}}\approx 0.5\right)$ into the gas cell (at a pressure of 760 Torr). By tracking the changes in the activity and structure of the NPs from 250 to 600 °C, we found that the NPs are inactive up to 250 °C, and the activity drastically increases at 300 °C and does not change significantly with further temperature increase (Figure 2B). Here, we note that the relatively high activation temperature in our operando TEM experiment can be attributed to the short gas residence time at the catalyst surface.^[^ ^10^ ^]^ The structural changes taking place during the reaction can be understood from the NP images at the onset of the reaction (*T *= 300 °C) and at the slightly elevated reaction temperatures (*T* = 400 °C) shown in Figure 2C. At the onset of the reaction, the solid Ni core starts to hollow and hollowing stops at 400 °C. Furthermore, at 400 °C, the overall size of the Ni core increased while its contrast in the image decreased, indicating that some of the Ni might have evolved into NiO. We confirmed this Ni to NiO transition by inspecting the high‐resolution images of Ni–Rh NPs and their fast Fourier transform (FFTs) at 250 and 400 °C (Figure 2D). Furthermore, STEM‐EDX images shown in Figure 2F, also confirm that the solid Ni core of the NPs transforms into a hollow NiO core still surrounded by Rh NPs during the CO oxidation reaction (see Figure S2, Supporting Information, for more image). Hence, both the morphology and composition of Ni–Rh NPs have been changed during the CO oxidation reaction.

To verify that the Ni to NiO transformation occurred for all the NPs, we acquired electron diffractions at 250 and 400 °C from a larger area containing many NPs, as shown in Figure 2E. At 250 °C, the diffraction rings correspond to {111}, {200}, and {220} planes of metallic Ni, whereas at 400 °C, two new rings that correspond to {111} and {220} planes of NiO appeared (Figure 2E) (see Figure S3, Supporting Information, for additional details). Furthermore, we also show the transformation of Ni to NiO by performing X‐ray photoelectron spectroscopy (XPS) analysis on as‐synthesized and postreaction NPs (Figure S4, Supporting Information).

**Figure 3:**
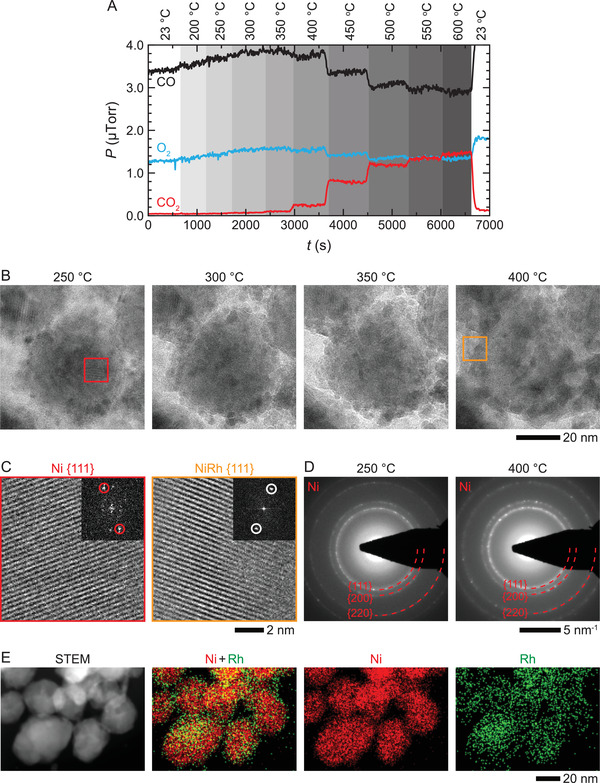
Operando TEM of Ni–Rh NPs during CO oxidation under O_2_‐poor condition. A) Changes in gas compositions during the CO oxidation reaction at different temperatures under 760 Torr of 28% CO, 14% O_2_, and 58% He gas environment, which corresponds to a gas pressure ratio of $p_{\mathrm{CO}}/p_{O_{2}}\approx 2.0$. B) Sequence of TEM images of Ni–Rh NPs at elevated temperatures and under the gas environment described in (A). C) High‐resolution TEM images from the areas selected by red and orange boxes in (B). Insets are the corresponding FFT patterns. The lattice spacing of *d*_Ni {111}_ =  0.20 nm changed to *d*_NiRh {111}_ =  0.21 nm when the temperature was increased from 250 to 400 °C. D) Electron diffractions patterns of Ni–Rh NPs at 250 and 400 °C, and dashed red quarter rings correspond to Ni. Note that because of an extremely small amount of Rh, diffraction rings associated with NiRh are absent. E) STEM image and corresponding EDX chemical maps of Ni–Rh NPs after the reaction at 400 °C.

Next, in a similar fashion, we tested how the Ni–Rh NPs behave under O_2_‐poor conditions by introducing a gas mixture comprising 28% CO, 14% O_2_, and 58% He ($p_{\mathrm{CO}}/p_{O_{2}}\approx 2.0$) into the gas cell. Here, the NPs remain inactive up to 350 °C and show a gradual increase in activity starting from 400 to 600 °C (**Figure** **3**A). To understand structural change taking place during this reaction, we analyzed the NPs at the onset of the reaction (*T *= 400 °C). Figure 3B reveals that the contrast of Rh NPs in the image series decreases gradually as the temperature is increased above 250 °C and the overall contrast of the NP becomes more uniform at 400 °C, which coincides with detectable CO conversion. The reason for disappearing contrasts of the Rh NPs in the image series is most likely due to their alloying with the Ni core to produce a NP with NiRh‐alloy surface. To confirm the formation of NiRh‐alloy, we inspected the high‐resolution images of Ni–Rh NPs and corresponding FFTs at 250 and 400 °C (Figure 3C). These images show that the lattice spacing changes from 0.20 nm (corresponding to Ni {111}) at 250 °C to 0.21 nm (corresponding to NiRh‐alloy {111}) at 400 °C.^[^ ^27^, ^28^ ^]^ These conclusions are also supported by our STEM‐EDX showing the Ni–Rh NPs transforming into a NP with the Ni‐core and NiRh‐alloy shell during the reaction (Figure 3E). It is worth noting that in this O_2_‐poor environment, NiO is absent, and we verified this by obtaining electron diffraction patterns at 250 and 400 °C from a larger area containing many NPs, which do not display any rings associated with NiO (Figure 3D) (see Figures S5 and S6, Supporting Information, for additional details).

Thus, unlike in an O_2_‐rich environment, which produces NiO core (Figure 2), the conversion reaction in an O_2_‐poor environment results in the NP with NiRh‐alloy surface. The lower activation temperature under the O_2_‐rich environment can be attributed to the presence of (Ni+NiO)–Rh surfaces. We confirmed that the trends in activities under different environments observed in operando TEM hold true when the reaction is conducted in a conventional reactor (Figure S7, Supporting Information).

Note that the same NPs transform into two different structures (Figure 2 vs Figure 3) under O_2_‐rich and O_2_‐poor conditions suggesting the composition of a reactive environment is the key driver of the observed structural changes.

To remove any doubt that the observed structural changes may be a result of thermal effects or related to the electron beam used for imaging the reactions, we conducted a series of control experiments. First, we imaged the final products, both under O_2_‐rich and O_2_‐poor conditions in our gas cell, that were not exposed to the electron beam (Figures S8 and S9, Supporting Information), and found their structures to be identical to those shown in Figures 2 and 3. Second, we investigated the evolution of Ni–Rh NPs by heating them under vacuum. The EDX and electron diffraction images show that the Ni–Rh NPs, for the most part, maintain their structures with the exception of a few of Rh NPs aggregated (Figure S10, Supporting Information). Hence, all the transformations observed under O_2_‐rich and O_2_‐poor conditions are because of the reactive gas environment.

**Figure 4:**
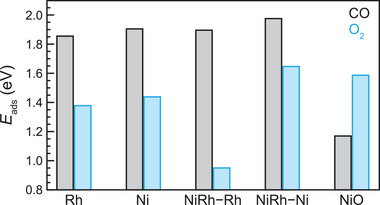
Density functional theory (DFT) based calculations of the adsorption energies of CO and O_2_ molecules on five different surfaces: Ni {111}, Rh {111}, NiO {111}, NiRh with an exposed Rh layer, and NiRh with an exposed Ni layer (Tables S1–S3, Supporting Information).

To explain the origin of low‐temperature activity of the Ni–Rh nanocatalysts in an O_2_‐rich environment, we evaluated the adsorption energies of CO and O_2_ onto (Ni+NiO)–Rh, Ni, Rh, and NiRh‐alloy surfaces using density functional theory (DFT) calculations (Figure S11, Supporting Information). By comparing the relative adsorption energy (i.e., coadsorption competition) between O_2_ and CO, one can estimate the CO tolerance of a catalyst.^[^ ^29^ ^]^ From computed adsorption energies (**Figure** **4**), we find that adsorption of CO onto Rh {111}, Ni {111}, and NiRh {111} surfaces is more preferred than the adsorption of O_2_, indicating that these surfaces are prone to CO‐poisoning (i.e., lower catalytic activity). In contrast, O_2_ adsorbs more readily than CO onto a NiO {111} surface (Figure 4), which is present only under O_2_‐rich conditions, indicating the absence of CO‐poisoning. Hence, Ni–Rh NPs (with NiRh‐alloy surface under O_2_‐poor environment) (Figure 3) along with pure Ni and pure Rh NPs are inactive at low temperatures because of CO poisoning, whereas Ni–Rh NPs (with (Ni+NiO)–Rh surface configuration under O_2_‐rich environment) exhibit high activity (Figure 2 and Figure S7, Supporting Information). Note that our results establishing Rh–NiO interface as an active site is consistent with recent studies of PtNi bimetallic nanocatalysts, which also showed Pt–NiO*_x_* interface to be responsible for their high catalytic activity.^[^ ^30^, ^31^ ^]^

It is worth mentioning here that earlier studies attributed the enhancement in the activity of bimetallic catalysts to their alterable electronic structures^[^ ^32^, ^33^ ^]^ while ignoring their structural transformations. In these earlier studies, the role of the structure was overlooked due to the lack of suitable approaches that enable direct nanoscale visualization of the reactions. Our study reveals that dynamic structural transformations that take place during the reaction are critical for catalysts′ performance, thus, highlighting the importance of operando TEM studies in understanding catalytic reactions.

## 3 Conclusions

In conclusion, by leveraging on synergetic effect between Ni and Rh, we synthesized high‐performance Ni–Rh nanocatalysts for a CO oxidation reaction. Direct observations of the reactions revealed that the structure of the NPs, and their corresponding activity, are dictated by the reactive environment during the reaction. Our results highlighting the multiple Rh–NiO interfaces as the main culprit for the observed low‐temperature activity show the importance of nanoscale visualization of chemical processes for understanding key parameters that drive these processes. We believe that studies like ours will be critical not only for the design of future catalysts but can be extended to a wide range of other gas‐based reactions.

## 4 Experimental Section

### Chemical Reagents

The following reagents were used in this study: rhodium(III) acetylacetonate (Rh(acac)_3_) (Cat. No. 282774, Sigma‐Aldrich Co., St Louis, MO, USA), nickel(II) acetylacetonate (Ni(acac)_2_) (Cat. No. 283657, Sigma‐Aldrich Co., St Louis, MO, USA), polyvinylpyrrolidone (PVP) with a molecular weight of 8000 (Cat. No. 41626, Alfa Aesar, Haverhill, MA, USA), ethylene glycol (EG) (Cat. No. 324558, Sigma‐Aldrich Co., St Louis, MO, USA), ethanol (Cat. No. E7023, Sigma‐Aldrich Co., St Louis, MO, USA). These reagents were used as received without further purification. Solutions were prepared using deionized water with a resistivity of 18.2 MΩ cm.

### Synthesis of Ni–Rh Heterostructured NPs

The bimetallic Ni–Rh heterostructured NPs shown in Figure 1A–D were synthesized through one‐pot synthesis method. Here, 10 µmol Rh(acac)_3_, 25 µmol Ni(acac)_2_, and 75 mg of PVP were added to a flask containing 15 mL of EG. The resulting light yellow mixture was heated at 100 °C until it became a homogenous solution. The obtained solution was then heated in an oil bath at 180 °C for 10 min to produce a black suspension. After cooling down to room temperature, the product NPs were obtained by filtering the solution, washing the NPs in ethanol, and then drying them at 60 °C for 2 h. The samples were further dispersed in deionized water by ultrasonication. 1 µL of this solution was dropcasted on the heater area of a microfabricated chip of the gas cell for operando TEM studies.

### Operando TEM Experiments

An in situ gas‐phase TEM imaging platform was used where the Ni–Rh heterostructured NPs were encapsulated within a microfabricated gas cell with a thin film heater (Figure 2A). Each gas cell consists of two chips with SiN*_x_* membrane windows, which sandwich NPs to form a sealed gas micro‐chamber. The SiN*_x_* window of the bottom chip contains a thin‐film Mo heater. The gas cell is loaded into a Climate in situ TEM holder, which has an inlet and outlet connected to a gas delivery system and mass spectrometer, respectively (DENSsolutions, Delft, Netherlands).^[^ ^34^ ^]^ In our experiments, the gas cell was maintained at ambient pressure (760 Torr). The TEM image series were acquired in Thermo Fisher 300 kV Titan TEM equipped with a Gatan K2 IS direct electron detection TEM camera (Gatan Inc., Pleasanton, CA, USA). In these experiments, we optimized the imaging conditions such that the electron flux was kept < 100 e^−^ Å^−2^ s^−1^ at all times to avoid electron beam‐induced artifacts, which is lower than what is commonly used in similar high resolution in situ TEM studies.^[^ ^10^, ^15^, ^35^ ^]^

For operando TEM experiments, the NPs were heated to 300 °C under a flow of Ar for 30 min followed by heating under CO atmosphere at 300 °C for 15 min. The gas composition was adjusted by changing the gas flow in individual mass flow controllers installed within the gas delivery system (DENSsolutions, Delft, Netherlands). Here, two gas cylinders were used, one with pure CO and one with a premixed gas comprising 20% O_2_ and 80% He. To increase the reaction output, a slow flow rate of 0.08–0.10 mL min^−1^ in the gas cell was used. The inline gas analyzer (DENSsolutions, Delft, Netherlands) was connected to the holder outlet line and the gas compositions were measured with a quadrupole mass spectrometer (Stanford Research Systems, Sunnyvale, CA, USA). The amount of gas going into the analyzer chamber was controlled by a leak valve so that the chamber pressure was maintained in the range of 10^−5^ Torr.

### Measurement of Catalytic Activity

The catalytic activity measurements displayed in Figure 1E and Figure S7 in the Supporting Information were carried out at ambient pressure (760 Torr) in a continuous flow fixed‐bed quartz tubular reactor. For the O_2_‐rich condition, the reactant gas mixture consisted of 1.0% CO, 2.0% O_2_, balanced with Ar. In the case of O_2_‐Poor condition, the reactant gas mixture consisted of 2.0% CO, 1.0% O_2_, balanced with Ar. Gases were mixed by the mass flow controllers and passed through the quartz tubular reactor at a fixed flow rate of 40 mL min^−1^. 5 mg of Ni–Rh heterostructured NPs, pure Ni, and pure Rh NPs were mixed with 1000 mg of SiO_2_ powder (Cat. No. S861672, Macklin Co., Shanghai, China), respectively. To remove the possible metal impurities, the SiO_2_ powder was washed with 1.0% v/v HNO_3_ solution prior to the mixing. The catalysts were loaded into the middle of the quartz tubular reactor and immobilized with quartz wool and were heated in a ceramic furnace. To obtain the light‐off curves, the catalysts were heated to the desired temperatures, and reactants and products were analyzed by an inline gas chromatograph (GC‐14C, Shimadzu Co., Kyoto, Japan) equipped with a thermal conductivity detector, using a 13X packing column. The conversion rates shown in Figure 1E and Figure S7 in the Supporting Information were obtained by averaging three measurements.

### XPS

XPS measurements were performed using a Kratos X‐ray photoelectron spectrometer (Axis Ultra DLD) (Kratos Analytical Ltd, Manchester, UK) at the base pressure of 3 × 10^−9^ Torr with a monochromatic Al *K*_*α*_ (1486.6 eV) X‐ray as the excitation source. The analyzer pass energy was set at 160 eV for survey scans and 40 eV for high‐resolution scans. The sample was positioned perpendicular to the detector and kept at room temperature during the measurements. All of the XPS spectra were calibrated with the C 1s peak at 284.8 eV as the binding energy reference.

### DFT Based Calculations

Periodic DFT calculations were performed with the Quantum Espresso code.^[^ ^36^, ^37^ ^]^ The Perdew–Burke–Ernzerhof generalized gradient approximation was used for exchange‐correlation energy,^[^ ^38^ ^]^ and projector augmented wave method pseudopotentials from PSlibrary (a library of pseudopotentials) were used to represent ion–electron interactions.^[^ ^39^, ^40^ ^]^ The electronic configuration for C, O, Rh, and Ni atoms are 2s^2^2p^2^, 2s^2^sp^4^, 5s^2^5p^0^4d^7^, and 4s^2^4p^0^3d^8^, respectively. We set the cutoff energy for the plane waves to 54 Ry (734 eV). The conjugate‐gradient‐like diagonalization algorithm was used, with a convergence threshold of 10^−6^ Ry. The Marzari–Vanderbilt–DeVita–Payne cold smearing with a width of 0.13 eV was used.^[^ ^41^ ^]^ The optimization process was carried out for all the structures until the force on the atoms dropped below 27 meV Å^−1^. Ni {111}, Rh {111}, and NiRh {111} surfaces were modeled by *p*(2  ×  2) four‐layer slab with a vacuum layer of 12 Å. An eight‐layer reconstructed *p*(2  ×  2) O‐terminated octopolar NiO {111} surface with a vacuum gap of 12 Å was chosen to represent NiO surface.^[^ ^42^, ^43^ ^]^ A ferromagnetic and antiferromagnetic phases were adopted for Ni {111} and NiO {111} surfaces, respectively. The Monkhorst–Pack method was used to sample the reciprocal space.^[^ ^44^ ^]^ The adsorption energies were evaluated as Δ*E*  = *E*_sub_  + *E*_mol_ − *E*_int_, where *E*_sub_, *E*_mol_, and *E*_int_ are the total energies of the substrate, the gas‐phase adsorbate molecule, and the interface, respectively.

## Conflict of Interest

The authors declare no conflict of interest.

## Supporting information

Supporting Information

## Data Availability

The data that support the findings of this study are available from the corresponding author upon reasonable request.
