# Supplementary material for: Revealing the Origin of Low‐Temperature Activity of Ni–Rh Nanostructures during CO Oxidation Reaction with Operando TEM
Source: Adv Sci (Weinh). 2022 May 5;9(17):2105599. doi: 10.1002/advs.202105599 (PMC9189651; doi:10.1002/advs.202105599)
Supplement: Supplementary file 1 — Supporting Information [file ADVS-9-2105599-s001.pdf]

ADVANCED SCIENCE
Open Access

Supporting Information

for *Adv. Sci.*, DOI 10.1002/advs.202105599

Revealing the Origin of Low-Temperature Activity of Ni–Rh Nanostructures during CO Oxidation Reaction with Operando TEM

*Tanmay Ghosh, Xiangwen Liu, Wenming Sun, Meiqi Chen, Yuxi Liu, Yadong Li and Utkur Mirsaidov**

ADVANCED SCIENCE
Open Access

Supporting Information

for *Adv. Sci.*, DOI 10.1002/advs.202105599

Revealing the Origin of Low-Temperature Activity of Ni–Rh Nanostructures during CO Oxidation Reaction with Operando TEM

*Tanmay Ghosh, Xiangwen Liu, Wenming Sun, Meiqi Chen, Yuxi Liu, Yadong Li and Utkur Mirsaidov**

Supporting Information

Revealing the Origin of Low-Temperature Activity of Ni–Rh Nanostructures during CO Oxidation Reaction with Operando TEM

Tanmay Ghosh[1, 2, †], *Xiangwen Liu*[1, 2, 3 †], *Wenming Sun*[4], *Meiqi Chen*[5], *Yuxi Liu*[5], *Yadong Li*[6], *and Utkur Mirsaidov*[1, 2, 7, 8*]

1. Department of Physics, National University of Singapore, Singapore 117551, Singapore
2. Centre for BioImaging Sciences, Department of Biological Sciences, National University of Singapore, Singapore 117557, Singapore
3. Institute of Analysis and Testing, Beijing Academy of Science and Technology (Beijing Center for Physical and Chemical Analysis), Beijing 100094, China
4. College of Science, China Agricultural University, Beijing 100193, China
5. College of Environmental and Energy Engineering, Beijing University of Technology, Beijing 100124, China
6. Department of Chemistry, Tsinghua University, Beijing 100084, China
7. Centre for Advanced 2D Materials and Graphene Research Centre, National University of Singapore, Singapore 117546, Singapore
8. Department of Materials Science and Engineering, National University of Singapore, Singapore 117575, Singapore

† These authors contributed equally to this work.

* Correspondence: mirsaidov@nus.edu.sg

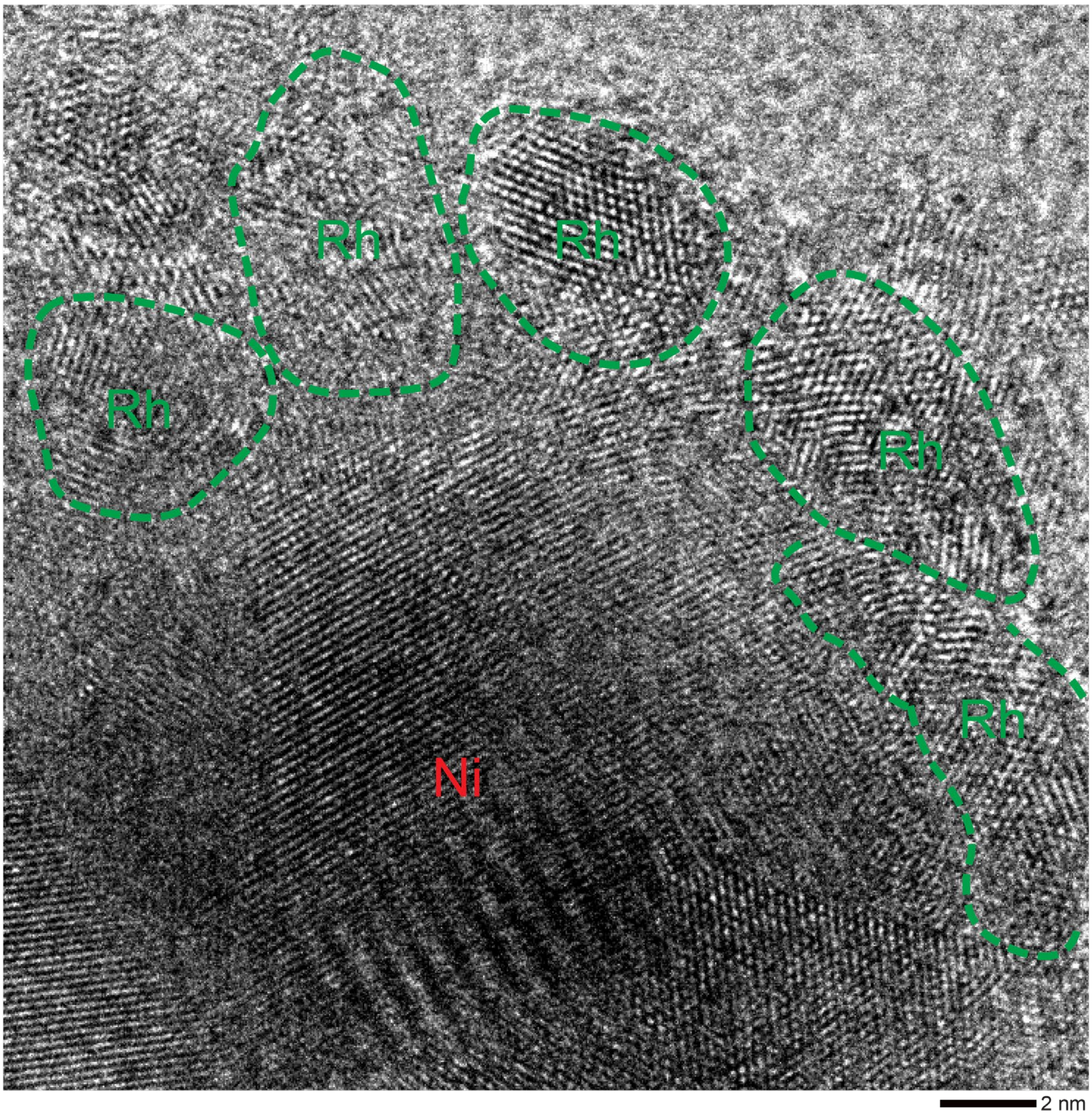

Figure S1. Ni–Rh heterostructured NPs. High-resolution TEM image of a corner of the Ni–Rh NP shown in Figure 1B, which confirms the crystallinity of a Ni core and small peripheral Rh NPs.

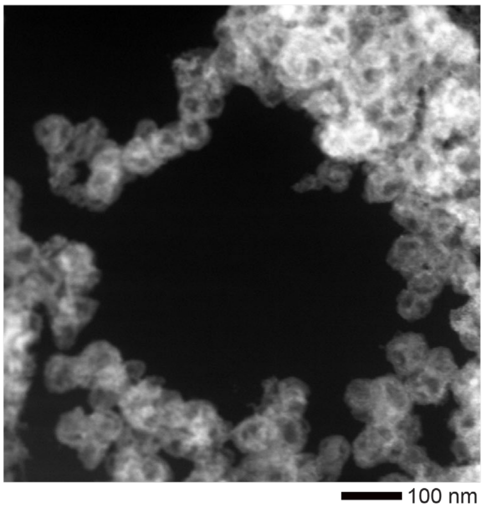

Figure S2. STEM image of Ni–Rh heterostructured NPs in O_2-rich environment. *In situ* STEM image of Ni–Rh NPs at 400 °C under an O_2-rich environment ($p_{CO}/p_{O_2} \approx 0.5$). All of the Ni–Rh NPs have transformed into hollow NPs with NiO shells. The smaller Rh NPs are still on the surfaces of their hollow structures, as seen in Figure 2F.

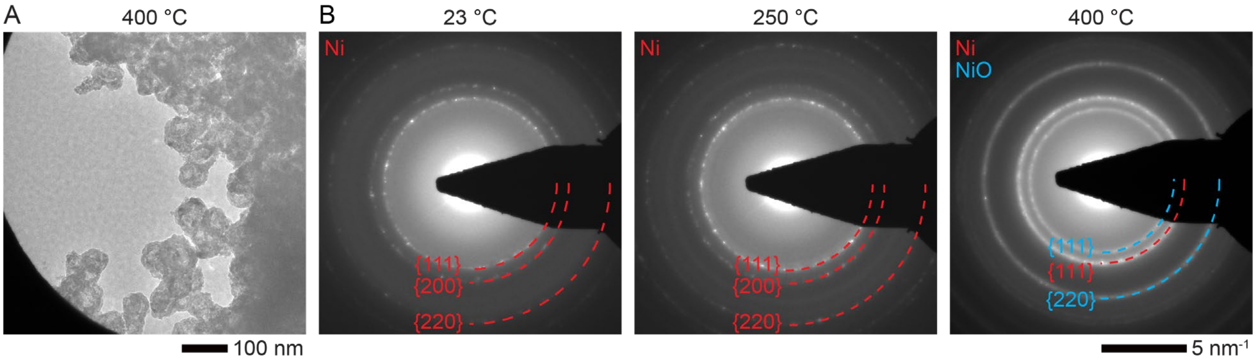

Figure S3. Electron diffraction of Ni–Rh heterostructured NPs in O_2-rich environment. (**A**) Selected area containing many NPs from which the *in situ* electron diffraction patterns have been acquired. (**B**) Selected area electron diffraction (SAED) images at 23, 250, and 400 °C display diffraction rings associated with {111}, {200}, and {220} planes of Ni at all temperatures and {111} and {220} planes of NiO at 400 °C. The presence of NiO rings suggests that some Ni transforms into NiO during the CO oxidation reaction in an O_2-rich environment.

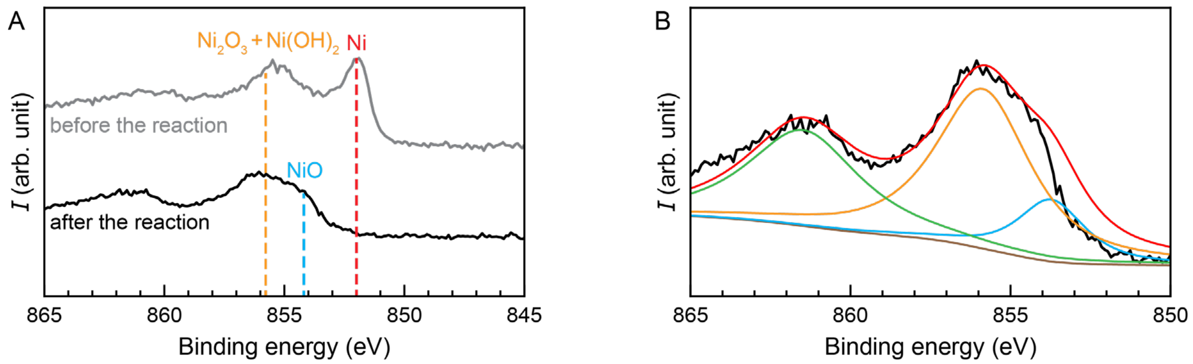

Figure S4. X-ray photoelectron spectroscopy (XPS) analysis of Ni–Rh heterostructured NPs. (**A**) XPS analysis of Ni–Rh NPs before and after CO oxidation under an O_2-rich environment (p_{CO}/p_{O_2} ≈ 0.5). Metallic Ni peak disappears, and the NiO peak appears after the reaction. This is consistent with the results from our operando TEM experiments shown in Figure 2. The peak at approx. 856 eV in both before and after the reaction spectra is most likely due to the presence of native nickel oxide (Ni_2O_3) and/or nickel hydroxide ($Ni(OH)_2$) that may form under a slightly humid environment.[1] (**B**) XPS of Ni–Rh NP after the reaction obtained with slightly higher resolution. Red, orange, blue, green, and brown color curves correspond to fits for Ni, Ni_2O_3 + $Ni(OH)_2$, NiO, satellite for nickel oxide, and the background signals, respectively.

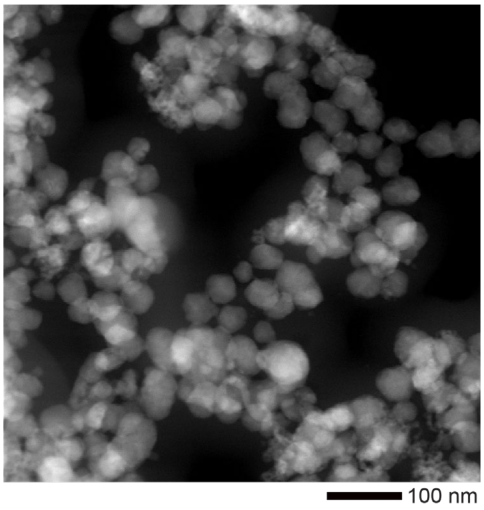

Figure S5. STEM image of Ni–Rh heterostructured NPs under O_2-poor conditions. *In situ* STEM image of Ni–Rh NPs at 400 °C under an O_2-poor environment (p_{CO}/p_{O_2} ≈ 2.0). All of the Ni–Rh NPs have transformed into smooth NPs with Ni core and NiRh-alloy shell, as shown in Figure 3E.

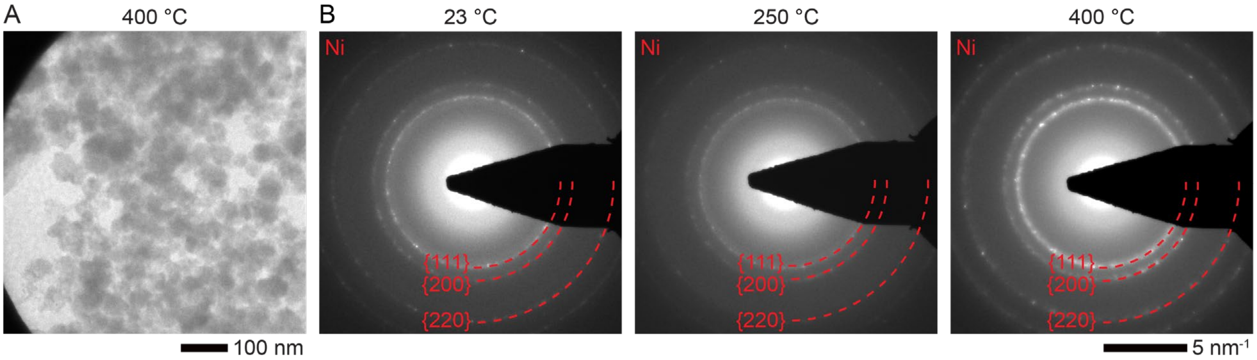

Figure S6. Selected area electron diffraction from Ni–Rh heterostructured NPs in O_2-poor environment. (**A**) Selected area containing many NPs from which the *in situ* electron diffraction patterns have been acquired. (**B**) SAED images at 23, 250, and 400 °C display diffraction rings associated with {111}, {200}, and {220} planes of Ni. The absence of rings associated with NiO suggests that no NiO forms during the CO oxidation reaction in an O_2-poor environment.

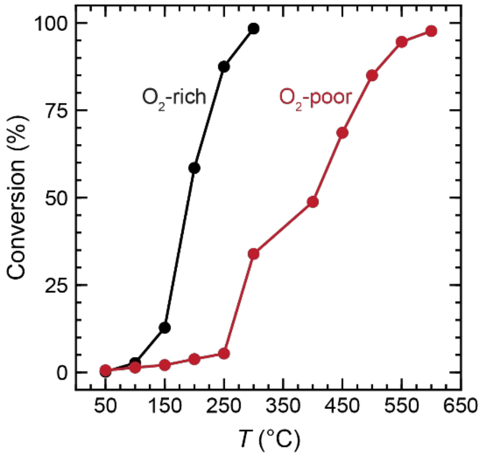

Figure S7. Activity of Ni–Rh heterostructured NPs towards CO oxidation reaction. CO oxidation activity of Ni–Rh NPs in O_2-rich and O_2-poor environments measured using a conventional fixed-bed reactor. In an O_2-rich environment, the NPs show ~95% CO conversion at around 300 °C, which is consistent with our operando TEM studies where the high conversion is seen at 300 °C (Figure 2B). In an O_2-poor environment, the NPs show ~90% CO conversion at around 550 °C, which is again consistent with our operando TEM studies where the high conversion is seen only at 600 °C (Figure 3A). These measurements confirm the upward shift in inactive-to-active transition temperature when we shift from an O_2-rich to an O_2-poor environment.

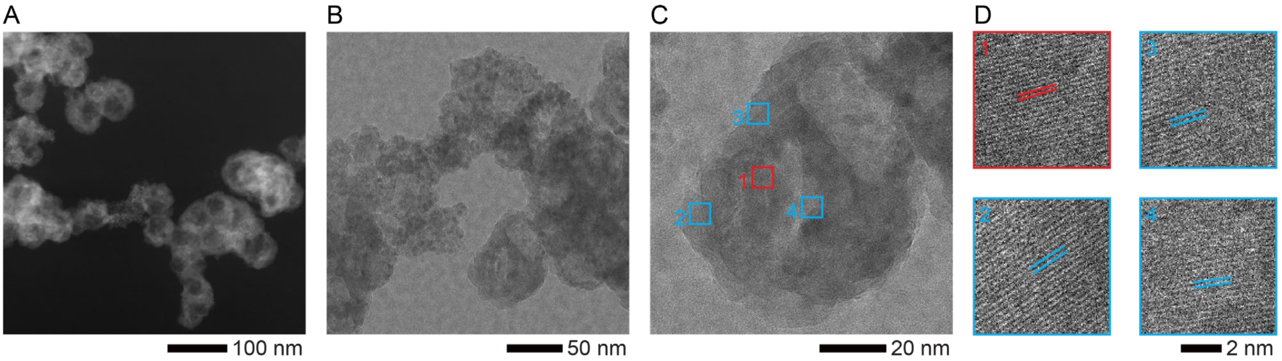

Figure S8. TEM images of Ni–Rh NPs from the area not exposed to electron beam during the CO oxidation reaction in an O_2-rich environment. (**A**) STEM image of Ni–Rh NPs at 450 °C. (**B**) Low and (**A**) high magnification TEM images of Ni–Rh heterostructured NPs in an O_2-rich environment ($^{p_{CO}}/_{p_{O_2}} \approx 0.5$) at 450 °C. (**D**) High-resolution TEM images from the areas selected by blue and red boxes in (C). Blue and red lines indicate the spacings between the lattice planes corresponding to $d_{\mathrm{Ni}\,\{111\}} = 0.20$ nm and $d_{\mathrm{NiO}\,\{111\}} = 0.24$ nm, respectively.

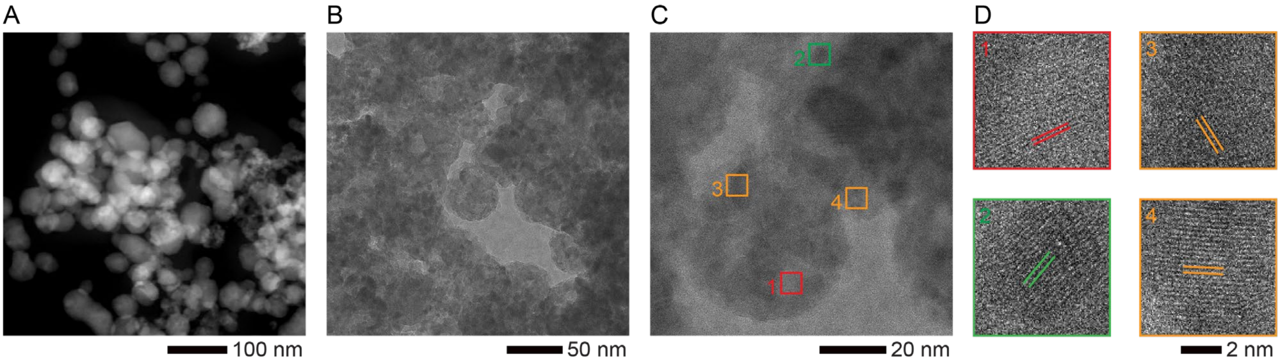

Figure S9. TEM images of Ni–Rh NPs from the area not exposed to electron beam during the CO oxidation reaction in an O_2-poor environment. (**A**) STEM image of Ni–Rh NPs at 500 °C. (**B**) Low and (**C**) high magnification TEM images of Ni–Rh heterostructured NPs in an O_2-poor environment ($^{p_{CO}}/_{p_{O_2}} \approx 2.0$) at 500 °C. (**D**) High-resolution TEM images from the areas selected by red, green, and orange boxes in (C). Red, green, and orange lines indicate the spacings between the lattice planes corresponding to $d_{\mathrm{Ni}\,\{111\}} = 0.20$ nm, $d_{\mathrm{Rh}\,\{111\}} = 0.22$ nm, and $d_{\mathrm{NiRh}\,\{111\}} = 0.21$ nm, respectively.

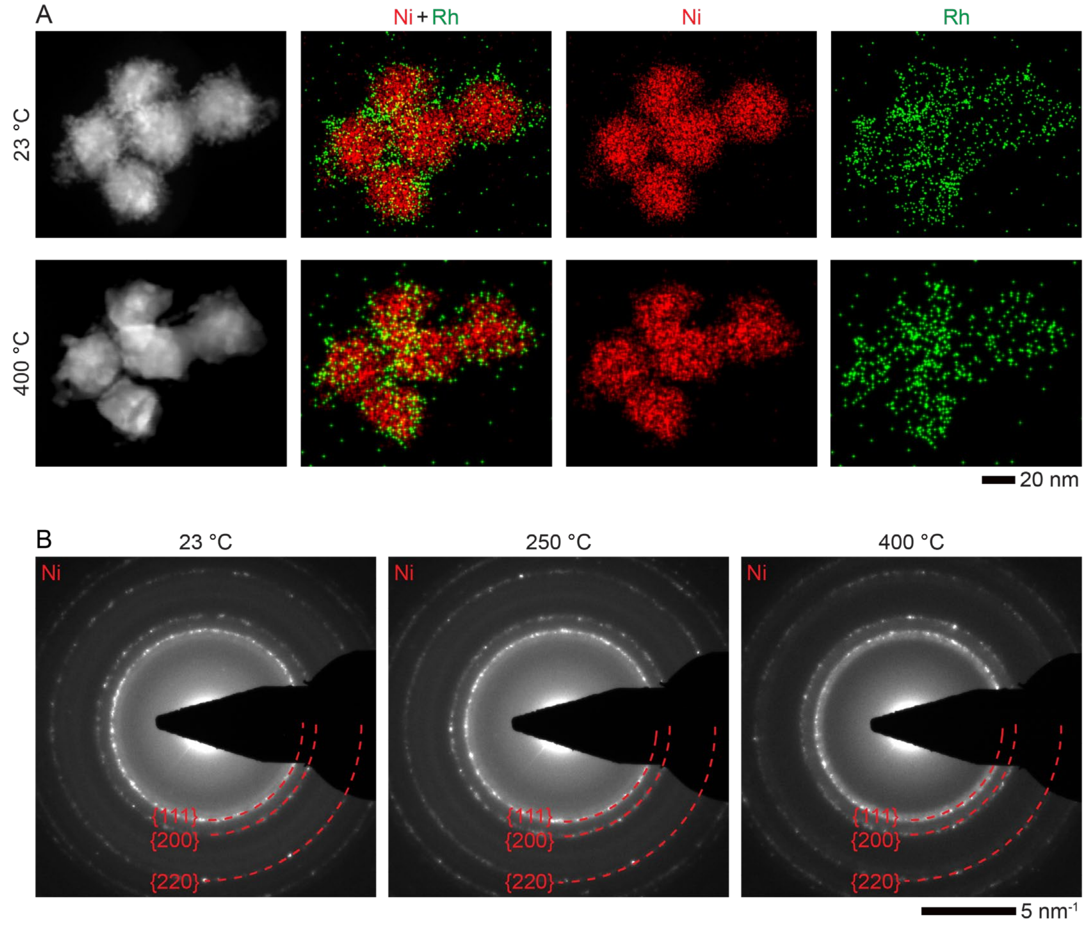

Figure S10. Heating Ni–Rh NPs under vacuum. (**A**) STEM images and corresponding EDX elemental maps of Ni–Rh NPs under vacuum at 23 and 400 °C. The STEM images do not display any apparent changes in the Ni–Rh NPs, with the exception of minor aggregation of a few Rh NPs on the surface of Ni cores. (**B**) *In situ* SAED images at 23, 250, and 400 °C display diffractions rings associated with {111}, {200}, and {220} planes of Ni.

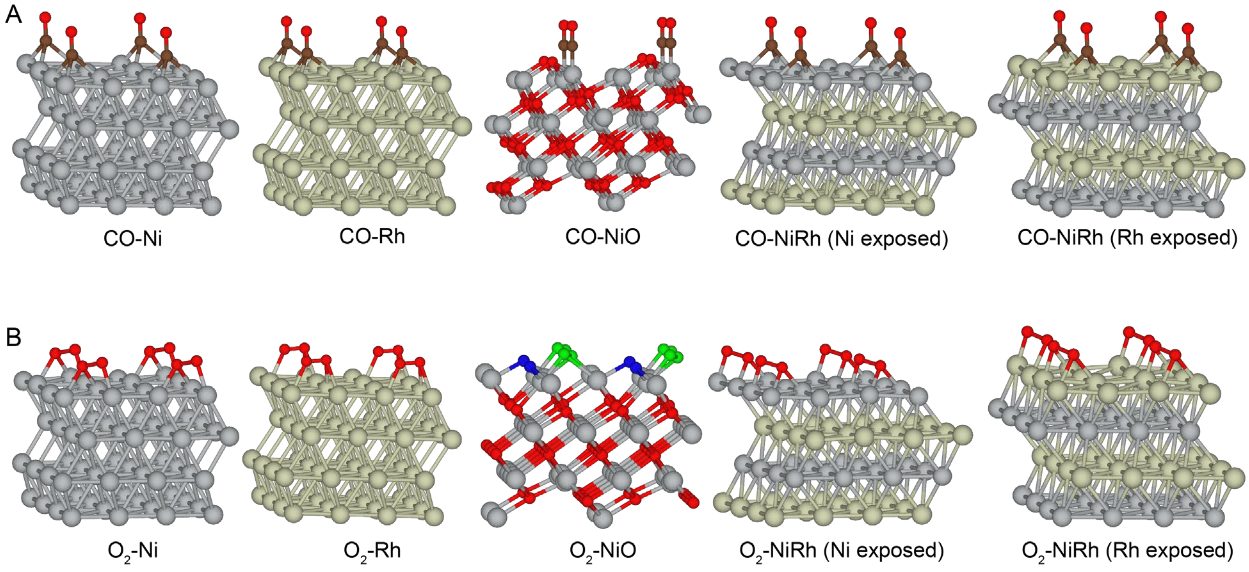

Figure S11. The adsorption configurations of CO and O_2 on the different surfaces. (**A**) Modeled structure showing CO molecules on the Ni, Rh, NiO, and NiRh alloy {111} surfaces. (**B**) Modeled structure showing O_2 molecules on the Ni, Rh, NiO, and NiRh alloy {111} surfaces. The atoms are represented as spheres: C (brown), Ni (grey), Rh (yellow), and O (red). Green and blue spheres represent the adsorbed oxygen molecules and lattice oxygen atoms in O_2–NiO model, respectively.

Table S1. Bond lengths between adsorbate molecules and metal/alloy surfaces. On these surfaces, CO adopts a vertical adsorption configuration while O_2 adopts a nearly parallel configuration. Hence, for CO, only the distances between C and its neighboring metal (M) atom are listed. For O_2, only the three shortest O–M bonds are listed.

	D (Å)											
surface	Rh {111}			Ni {111}			NiRh {111}–Rh			NiRh {111}–Ni		
CO	2.093	2.099	2.103	1.943	1.946	1.948	2.074	2.075	2.077	1.954	1.956	1.956
O_2	1.990	2.271	2.307	1.831	1.913	1.914	1.934	2.115	2.116	1.816	1.910	1.911

Table S2. Bond lengths between adsorbate molecules and a NiO {111} surface. On this surface, CO adopts a vertical adsorption configuration while O_2 adopts a nearly parallel configuration. Hence, for CO, only the distances between C and its neighboring Ni atom are listed. For O_2, only the shortest of four O–Ni bonds are listed.

	D (Å)			
surface	NiO {111}			
CO	1.760			
O_2	2.013	1.877	2.238	1.854

Table S3. Adsorption energies of CO and O_2 onto various surfaces. Here, NiRh {111}–Rh and NiRh {111}–Ni represent the NiRh {111} surface with Rh and Ni layers exposed as the adsorbed sites, respectively.

	E_{ads}(eV)				
E_{ads}(eV)	Rh {111}	Ni {111}	NiRh {111}–Rh	NiRh {111}–Ni	NiO {111}
CO	1.86	1.91	1.90	1.98	1.17
O_2	1.38	1.44	0.95	1.65	1.59

Supplementary reference

[1] C.-L. Chang, S. K. R. S. Sankaranarayanan, D. Ruzmetov, M. H. Engelhard, E. Kaxiras, S. Ramanathan, *Phys. Rev. B* **2010**, 81, 085406.
